# High-Performance, Degradable, Self-Healing Bio-Based Nanocomposite Coatings with Antibacterial and Antioxidant Properties

**DOI:** 10.3390/nano13071220

**Published:** 2023-03-29

**Authors:** Qiang Zhang, Qihang Bu, Jiangyue Xia, Rongxue Sun, Dajing Li, Haibo Luo, Ning Jiang, Cheng Wang

**Affiliations:** 1 School of Food and Pharmaceutical Engineering, Nanjing Normal University, Nanjing 210023, China; 2 Institute of Agricultural Products Processing, Jiangsu Academy of Agricultural Sciences, Nanjing 210014, China; 3 Integrated Scientific Research Base for Preservation, Storage and Processing Technology of Aquatic Products of the Ministry of Agriculture and Rural Affairs, Nanjing 210014, China

**Keywords:** nanocomposite coating, self-healing, antioxidant, antibacterial

## Abstract

The purpose of this study is to obtain a bio-based coating with good functional activity and self-healing ability, demonstrating its potential in food, materials, and other application fields. Plastic coatings can cause serious environmental pollution. It was a good solution to replace plastic coatings with degradable coatings. However, the development of degradable coatings in the fields of food and materials was limited due to their insufficient antibacterial ability and weak comprehensive properties. Therefore, chitosan nanoparticles (NPs) loaded with gallic acid (GA) were self-assembled with gelatin (GE) to prepare high-performance, degradable, self-healing bio-based nanocomposite coatings with antibacterial and antioxidant properties. The oxygen permeability of GE nanocomposite coatings decreased gradually with the addition of NPs, and the barrier properties increased significantly. At the same time, due to the excellent antioxidant and antibacterial ability of GA, the antioxidant effect of the nanocomposite coatings increased by 119%, and the antibacterial rate against *Escherichia coli* (*E. coli*) and *Staphylococcus aureus* (*S. aureus*) increased by 32% and 58%, respectively, compared with the pure GE coatings. In addition, the nanocomposite coatings can be repaired within 24 h after being scratched at room temperature. Finally, GA coated with chitosan nanoparticles can significantly delay the escape of GA, and the retardation of gallic acid release exceeded 89% in simulated solutions after 24 h immersion, extending the service life of the nanocomposite coatings.

## 1. Introduction

Coatings had great application value in food, materials, and other fields. Such as delaying the spoilage of food, preventing the oxidation damage of materials, and so on [1,2]. Compared with non-degradable plastic coatings (such as polyethylene, polystyrene, and polycarbonate), degradable coatings had attracted extensive attention from researchers due to their unique biodegradability [3,4,5,6]. It was found that eco-friendly coatings (such as hydrolyzable polymers and bio-based coatings) had good degradability and biocompatibility, which can effectively reduce environmental pollution and alleviate increasingly prominent environmental problems [7]. However, eco-friendly coatings applications were limited due to the insufficient antibacterial effect and weak mechanical properties [8]. Therefore, it is particularly important to develop eco-friendly coatings with antibacterial effects. Adding antibacterial agents to the coating is one of the simple and efficient solutions. The purpose of killing bacteria was achieved by the bactericidal effect of antimicrobial agents. However, the traditional fungicides, such as silver ion and metal oxide had some problems of high toxicity and high cost [9]. By contrast, although natural antimicrobials had poor heat resistance and short antimicrobial duration, they were highly valued by researchers for their safety, non-toxicity, cost-effectiveness, and antioxidant properties. Zhao et al. [10] introduced berberine hydrochloride with broad-spectrum antibacterial activity into polyurethane fiber and found that the composite polyurethane fiber can inhibit the activity of respiratory chain hydrogenase in *Escherichia coli* (*E. coli*) and *Staphylococcus aureus* (*S. aureus*), thus inhibiting the growth of bacteria. At the same time, it can also destroy the bacterial cell wall and cell membrane, resulting in the leakage of intracellular macromolecules. Charlotte et al. [11] immobilized *Nisin* into ultra-thin hydration coatings, which showed strong inhibition against *S. aureus*. Zhu et al. [12] utilized a layer-by-layer technique to assemble tannic acid, gentamicin, and polymyxin B into a new antibacterial coating that significantly inhibited the growth of *Staphylococcus epidermidis* (*S. epidermidis*) and *E. coli*.

However, the antibacterial coatings can be damaged during transportation, storage, and use due to collision, friction, and other factors, which can reduce the protective properties of the antibacterial coatings. Therefore, it was imperative to develop antibacterial coatings with self-healing ability. The self-healing coating is a kind of coating that can repair the damage autonomously and restore its original structure and function. It can be divided into two types according to the mechanism of self-healing. The first repair method involved embedding microcapsules into the coating. When the coating was damaged, the active substance was released from the microcapsules to form a new protective barrier [13]. Song et al. [14] polymerized isophorone diisocyanate (IPDI) and 4, 5-dichloro-2-n-octyl-4-isothiazolin-3-one (DCOIT) into microcapsules and added them to the antibacterial coatings to make the antibacterial coatings unfold good self-healing properties. At the same time, in the absence of catalysts, the antibacterial properties of the self-healing coatings against *E. coli* and *P. aeruginosa* were still significant. However, although this type of repair method can restore the original structure of the coatings, the repair ability of the coatings was limited due to the limitations of the repairing agents and some differences from the coating itself [15,16]. By contrast, another alternative approach to self-healing through intramolecular forces such as host-guest interaction, ionic interaction, and the dynamic covalent bond can avoid the limitation of healing times [17,18,19]. Therefore, in recent years, this kind of restoration method received widespread attention from researchers at home and abroad. Guo et al. [20] prepared a high-strength self-healing polysiloxane containing N-acetyl-L-cysteine (NACL) side groups. The self-healing of the polysiloxane was realized by the hydrogen bond interaction of NACL. Meanwhile, the mechanical properties and antibacterial properties of polysiloxane coatings were significantly improved with the addition of NACL. Du et al. [21] prepared the self-healing coatings by layer-by-layer assembly of chitosan and sodium alginate, and realized the self-healing of the coatings by utilizing the hydrogen bonds and the fluidity of the chitosan chains in the coatings. At the same time, it was found that compared with the coatings that could not self-heal, the coatings that realized self-healing after damaged could still effectively delay the deterioration of strawberries.

In this study, high-performance, degradable, self-healing bio-based nanocomposite coatings with antibacterial and antioxidant properties were designed and prepared. Utilizing gallic acid (GA) as an antibacterial agent and sodium tripolyphosphate (TPP) as the cross-linking agent, chitosan (CS) nanoparticles (NPs) loaded with GA combined antibacterial and antioxidant activity were prepared by cross-linking CS with TPP. The NPs was added to the gelatin (GE) coatings to enhance the antibacterial and antioxidant properties of the GE coatings. At the same time, the hydrogen bond interaction between GE substrates was utilized to realize the self-healing of the coatings.

## 2. Materials and Methods

### 2.1. Materials

Chitosan (CS) and glacial acetic acid were purchased from Shanghai McLean Biochemical Technology Co., Ltd. (Shanghai, China). The average molecular weight of CS was 150 kDa and the degree of deacetylation was 85%. Sodium tripolyphosphate (TPP), gallic acid (GA), and gelatin (GE) were purchased from Shanghai Aladdin Biochemical Technology Co., Ltd. (Shanghai, China). Sodium hydroxide (NaOH) was purchased from Xilong Science Co., Ltd. (Shantou, China). 2,2′-Azino-bis (3-ethylbenzothiazoline-6-sulfonic acid) diammonium salt (ABTS) was purchased from Nanjing Middle East Chemical Glass Instrument Co., Ltd. (Nanjing, China). *Escherichia coli* (*E. coli*, ATCC25922) and *Staphylococcus aureus* (*S. aureus*, ATCC25933) were purchased from the American Type Culture Collection (ATCC) (Manassas, VA, USA).

### 2.2. Preparation of Coatings

**Figure 1:**
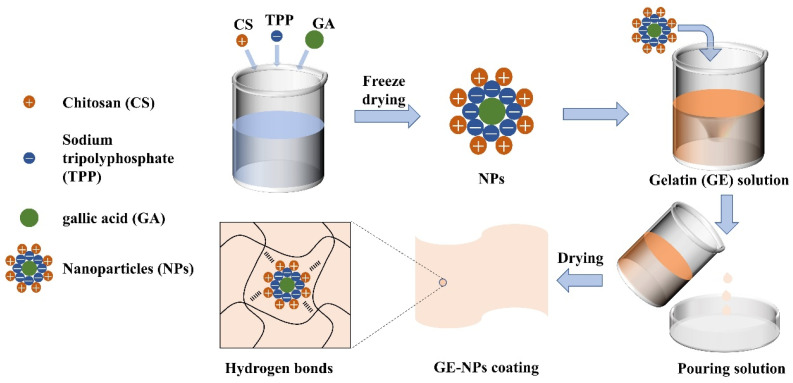
Schematic diagram of the preparation of GE nanocomposite coating.

The fabrication of GE coatings was determined according to Liu et al. [22]. As can be seen from Figure 1, CS was dissolved in 1% (*v/v*) acetic acid solution, GA was added, and then 10 M NaOH was added to adjust the Ph of CS solution (pH = 5). Under rapid stirring, TPP solution was added to the CS solution drop by drop to obtain NPs. NPs was freeze-dried at −55 °C for 48 h in a freeze dryer. 4% (*w/v*) GE was dissolved in ultrapure water, swelled at room temperature for 1 h, heated and stirred until the GE was completely dissolved, and 10% glycerin was added. The NPs were re-suspended and the GE-glycerol solution was added at the ratio of 3:2 to prepare coating solutions with different NPs contents (the coatings were named as GE, GE-NPs_2%_, GE-NPs_4%_, GE-NPs_6%_, GE-NPs_8%_, and GE-NPs_10%_, in which 2%, 4%, 6%, 8%, and 10% represented the weight ratio of NPs to GE, respectively). Thoroughly mixed, poured into plastic sheets (9 cm × 9 cm), and dried at 25 °C for 24 h. Before each test, keep a constant temperature and humidity (50% RH) for 48 h.

### 2.3. Structural Analysis

The infrared spectrum of the coatings was measured by a TENSOR Ⅱ FTIR spectrometer (BRUKER, Karlsruhe, Germany) with the wavelength range of 600 to 4000 cm^−1^ and a resolution of 2 cm^−1^. The X-ray diffraction spectra of the coatings were determined by an X-ray diffractometer (D2 PHASER, Bruker, Karlsruhe, Germany) and the geometry of the goniometer was the Bragg Brentano. The coatings (2 cm × 2 cm) were placed on a loading platform with 2θ ranging from 5° to 85°. The surface and cross-sectional morphology of the coatings were observed by a scanning electron microscope (EVO-LS10, Oberkochen, Germany). The sample was fixed and gold was sprayed. The thermal stability of the coatings was studied by a thermogravimetric analyzer (TG/DTA7200, Wilmington, DE, USA). The sample was weighed 2 mg and heated from 30 °C to 800 °C at a heating rate of 10 °C/min. Each sample was tested three times in parallel. The tensile strength and the elongation at break of the coatings were measured by an electronic universal testing machine (AGS-X-10 kN, Kyoto, Japan). The coatings were cut into rectangles (3 cm × 1 cm) and clipped on the fixture. The tensile rate was 100 mm/min.

### 2.4. Barrier Properties

The oxygen permeability of the coatings was measured by an oxygen transmittance tester (C230H, Jinan, China). The experimental temperature was set at 23 °C and the experimental humidity was set at 0% RH.

The light transmittance of the coatings was determined by a UV-visible spectrophotometer (M4PC, Shanghai, China), where the sample was inserted into the fixture and scanned in the range of 200–800 nm with a scanning interval of 1 nm.

### 2.5. Antioxidant Activity

The assay of antioxidant activity was adapted by Ge et al. [23]. 2.45 mM potassium persulfate and 7 mM ABTS were mixed in equal proportion and reacted in the dark for 12 h to produce ABTS free radicals. The ABTS solution was diluted properly so that the absorbance was 0.7 at the wavelength of 734 nm. The supernatant was added, reacted in the dark for 10 min, and then the absorbance was measured. The ABTS free radical scavenging rate of the sample was calculated by the following formula:

ABTS radical scavenging rate (%) = (A_0_ − A_1_)/A_0_ × 100% (1)

where A_0_ is the absorbance of ABTS and A_1_ is the absorbance of the sample solution. Each sample was measured three times in parallel, and the average value was taken as the final result.

### 2.6. Antibacterial Properties

The test of antibacterial properties was according to Xue et al. [24]. The activated *E. coli* and *S. aureus* (10^6^ CFU/mL) were coated on LB medium. The samples with diameters of 6 mm were pasted on the medium coated with bacterial solution after ultraviolet irradiation. The diameter of the bacteriostatic zone was measured after 24 h culture at 37 °C.

### 2.7. Release Assays

The experimental method of GA release was adapted by Roy et al. [25]. The GE-NPs_10%_ coating and GE-GA_10%_ coating (GE composite coating unencapsulated GA) were added to conical flasks containing different simulated solutions of 20 mL (50% alcohol, 95% alcohol, 3% acetic acid, and water to simulate different ethanol contents, acidic and neutral environments, respectively) and cultured in an oscillating incubator at 25 °C. The absorbance value of 1 mL sample solution was measured at a certain interval, and the same simulation solution was supplemented.

### 2.8. Statistical Analysis

All samples were measured at least three times, and the final results were expressed as average ± standard deviation. SPSS 26.0 software was used to analyze variance, and Duncan multiple comparisons were used to determine the significant difference (*p* < 0.05).

## 3. Results and Discussion

### 3.1. Fourier Transform Infrared (FTIR) Spectrum

**Figure 2:**
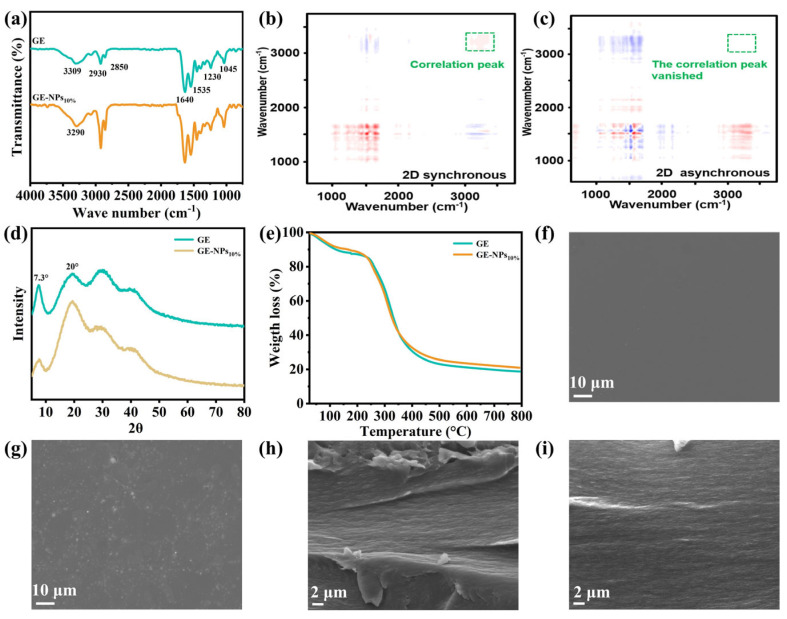
(**a**) FTIR spectrum of GE coating and GE-NPs_10%_ coating; (**b**,**c**) 2D correlation FTIR synchronous and asynchronous spectra of GE-NPs_10%_ coating; (**d**) XRD spectrum of GE coating and GE-NPs_10%_ coating; (**e**) TGA spectrum of GE coating and GE-NPs_10%_ coating; (**f**,**g**) SEM images of the surface of GE coating and GE-NPs_10%_ coating; (**h**,**i**) SEM images of the cross-section of GE coating and GE-NPs_10%_ coating.

FTIR can reveal the vibration information of functional groups and the interaction between molecules of the material. As shown in Figure 2a, in the spectrum of pure GE coating, the peak at 3309 cm^−1^ represented the stretching vibrations of -OH and -NH [26], while the peaks at 2930 cm^−1^ and 2850 cm^−1^ represented the asymmetric and symmetrical stretching vibrations of C-H, respectively [27]. The peaks at 1640 cm^−1^, 1535 cm^−1^, and 1230 cm^−1^ represented the stretching vibration of C=O in the amide Ⅰ band, the bending vibration of -NH in the amide Ⅱ band, and the stretching vibration of C-N in the amide Ⅲ band, respectively [28]. Besides, the peak at 1045 cm^−1^ represented the interaction between GE and the -OH in glycerol [29]. Due to the hydrogen bond interaction between the NPs and the GE matrix, the stretching vibration peak at 3309 cm^−1^ was red-shifted to 3290 cm^−1^ after the addition of NPs. At the same time, the nanocomposite coating did not produce new peaks, indicating that NPs and GE were physically bonded and did not form new chemical bonds [30]. In addition, two-dimensional correlation FTIR spectroscopy was used to prove the existence of hydrogen bond interaction. The presence of a typical cross-crossing peak of the interaction between hydroxyl and hydroxyl in Figure 2b,c shows the existence of intermolecular forces (hydrogen bonds).

### 3.2. X-ray Diffraction (XRD)

XRD can observe the microstructure and the degree of crystallization of the material. As shown in Figure 2d, the peak at 2θ = 7.3° was a characteristic peak of GE and represented the triple helix structure in the GE matrix [31]. The peak value of the characteristic peak is related to the diameter of the triple helix, and the peak intensity is related to the content of the triple helix. The characteristic peaks of the two samples in the figure did not deviate, indicating that they both had a constant triple helix diameter. However, the peak intensity decreased after the addition of NPs, indicating that the content of triple helix in the coatings decreased after the addition of NPs, which may be due to the hydrogen bond interaction between NPs and GE to reduce the crystallinity of the GE coatings [32]. The wide peak at 2θ = 20° represented the amorphous structure in the coatings. When the addition of NPs was 10%, the intensity of the diffraction peak increased and shifted to the right, representing the increase in the number of hydrogen bond interactions with GE after the addition of NPs [33]. In addition, the peaks at 2θ = 30° and 40° did not change after the addition of NPs, indicating that the addition of NPs did not change this part of the structure.

### 3.3. Thermogravimetric Analysis (TGA)

TGA can show the changes in materials with increasing temperature, and thus determine the thermal stability of materials. As can be seen from Figure 2e, the weight loss of the coatings can be divided into three stages. The temperature range of the first stage of weight loss was 25 °C to 180 °C, which was due to the mass loss caused by the evaporation of water in the coatings [34]. The temperature range of the second stage of weight loss was 180 °C to 280 °C, which was due to the mass loss caused by the degradation of glycerol and the decomposition of GA in the coatings [35]. The temperature range of the third stage of weight loss was 280 °C to 500 °C, which was due to the mass loss caused by the degradation of proteins in the GE coatings [36,37]. As shown in Table S1 and Figure S2, the initial decomposition temperature of the pure GE coating was 55 °C, while the decomposition temperature of GE-NPs_10%_ coating was 60 °C, which proved that the heat resistance of nanocomposite coatings was higher than pure GE coatings. Meanwhile, the weight loss rate of the nanocomposite coatings in the first and third stages was significantly lower than that of the pure GE coatings, which was due to the hydrogen bond interaction between NPs and GE, which can significantly enhance the heat resistance of the GE coatings. Therefore, the addition of NPs to GE coatings can effectively improve the thermal stability of GE coatings. However, in the second stage, the weight loss rate of the nanocomposite coatings was higher than pure GE coatings, which was due to the decomposition of GA in the nanocomposite coatings at this stage. The results showed that the addition of NPs to the pure GE coatings could effectively improve the heat resistance and significantly enhance the thermal stability of the GE coatings.

### 3.4. Scanning Electron Microscope (SEM)

The surface and cross-section morphology of the nanocomposite coatings were observed by SEM. As shown in Figure 2f, the pure GE coatings exhibited a smooth, uniform, bubble-free surface. The NPs were evenly distributed in the GE-NPs_10%_ coating (Figure 2g). In addition, the cross-section morphology of the GE coatings was shown in Figure 2h,i. There was little difference between the cross-section of the pure GE coatings and that of the nanocomposite coatings. All showed uniform and dense structures. The results showed that there was good chemical compatibility between the NPs and the GE matrix, and the NPs did not change the original structure of the pure GE coatings. The analysis of FTIR and XRD can support this conclusion.

### 3.5. Barrier Properties

#### 3.5.1. Oxygen Transmittance

**Figure 3:**
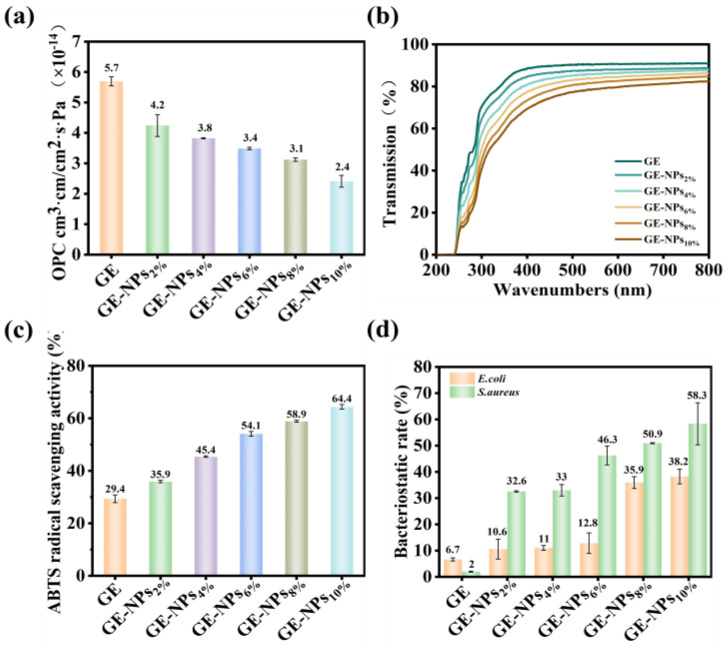
(**a**) OPC of GE coatings with different concentrations of NPs; (**b**) Light transmittance of GE coatings with different concentrations of NPs; (**c**) ABTS free radical scavenging rate of GE coatings with different concentrations of NPs; (**d**) Bacteriostatic rate of GE coatings with different bacteriostatic rate.

The oxygen barrier property is an important property of coatings in the field of materials. Improving the ability to block oxygen can effectively reduce the exchange of oxygen. As shown in Figure 3a and Figure S1, with the addition of NPs, the oxygen barrier property of the GE coating was gradually enhanced, and the oxygen permeability coefficient (OPC) decreased significantly, from 5.695 × 10^−14^ cm^3^ cm/cm^2^ s Pa to 2.409 × 10^−14^ cm^3^ cm/cm^2^ s Pa. This was due to the small size of NPs, which occupied the channels in the spatial network structure of GE and hindered the exchange of oxygen molecules, thus significantly increased the oxygen barrier property of the nanocomposite coatings [38]. In addition, the hydrogen bond interaction between NPs and GE caused the coating to become tighter, forming a dense structure that further prevented the exchange of oxygen molecules [30]. The results showed that adding NPs to the GE coatings can effectively reduce the oxygen permeability of the nanocomposite coatings, reduce the oxygen exchange, and enhance the oxygen barrier property of the GE coatings.

#### 3.5.2. Light Transmittance

Good UV-blocking properties can effectively reduce the photoinduced oxidation reaction. The light transmittance of the coatings gradually decreased with the addition of NPs between 200–800 nm (Figure 3b), and the UV transmittance of the coatings rapidly decreased to 0% between 200–400 nm. It was proved that the UV barrier properties of GE coatings can be further enhanced by adding NPs to pure GE coatings. This was because the benzene ring in GA has a strong UV absorption capacity [39], which can effectively absorb UV, and enhance the UV-blocking ability of the nanocomposite coatings. In addition, the absorption peaks at 259 nm and 279 nm were due to the strong UV absorption capacity of tryptophan, lysine, and phenylalanine in GE at 279 nm, 278 nm, and 259 nm, respectively [40]. The results showed that the addition of NPs to GE coatings can effectively reduce the UV transmittance, significantly improve the UV barrier properties of GE coatings, and avoid the occurrence of photoinduced oxidation.

### 3.6. Antioxidant Activity

The antioxidant activity of the GE coating was evaluated by ABTS free radical scavenging assays. As can be seen from Figure 3c, the antioxidant activity of the pure GE coating was the lowest. However, the antioxidant activity of GE nanocomposite coatings increased significantly with the addition of NPs. The ABTS free radical scavenging rate increased from 29.4% to 64.4% in a dose-dependent on NPs. The experimental results showed that the addition of NPs to GE coatings can significantly improve the antioxidant activity of GE coatings. Among them, the antioxidant activity of pure GE coatings was provided by the bioactive peptides produced by the hydrolysis of GE in water [41]. The significant enhancement of the antioxidant activity of the nanocomposite coatings was related to the CS and GA in the NPs. On the one hand, the free amino group in CS can absorb H^+^, to achieve the purpose of eliminating free radicals [42]. On the other hand, the hydrogen extraction reaction of the phenolic hydroxyl groups in GA can quench the free radicals and further scavenge the free radicals [43].

### 3.7. Antibacterial Properties

The antibacterial activity of GE coatings was reflected by antibacterial experiments against *E. coli* and *S. aureus*. As shown in Figure 3d, the antibacterial properties of pure GE coatings against *E. coli* and *S. aureus* were limited, and the inhibition rates were less than 10%. However, with the gradual increase of NPs, the antibacterial properties of GE nanocomposite coatings were significantly enhanced. This was due to the strong antibacterial properties of GA in NPs. GA can combine with the bivalent cation of the bacterial outer membrane to collapse the cell wall, destroy the integrity of the bacterial cell wall and the permeability of the cell membrane, resulting in the leakage of intracellular molecules, hinder the growth and reproduction of bacteria, and thus achieve the purpose of killing bacteria [44]. The results showed that the addition of NPs can effectively enhance the antibacterial properties of GE coatings.

### 3.8. Release Assays

**Figure 4:**
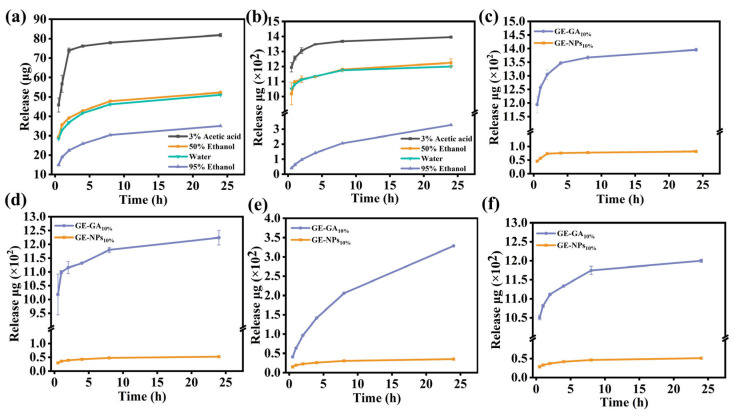
(**a**,**b**) Release assay of GE-NPs_10%_ coating and GE-GA_10%_ coating in simulated solution; (**c**–**f**) Release assay of GE-NPs_10%_ coating and GE-GA_10%_ coating in 3% acetic acid, 50% ethanol, 95% ethanol, and water.

The release assays of GA in different environments were studied by 50% ethanol, 95% ethanol, 3% acetic acid, and water as four simulated solutions (simulated different alcohol content, acidic and neutral environments, respectively). As shown in Figure 4a,b, the type of simulated solution had a significant effect on the release of GA in the GE nanocomposite coatings. The release rate of GA was the fastest in the 3% acetic acid solution, followed by 50% ethanol and water solution, and finally, the release rate was the slowest in the 95% ethanol solution. This was due to the good solubility of GE and CS in acetic acid solution, while the solubility in cold water is low [25]. At the same time, GE and CS are insoluble in ethanol, thus delaying the release of GA. Therefore, GA had the fastest release rate in the 3% acetic acid solution and the slowest release rate in the 95% ethanol solution. Meanwhile, since GE is hydrophilic and the solubility of GA in ethanol is higher than that in water, the release rate of GA in 50% ethanol solution is higher than that in water after GE hydrolysis. As can be seen from Figure 4c–f, the encapsulation of GA can significantly reduce the release rate of GA in the nanocomposite coatings in the four simulated solutions. On the one hand, this was because CS nanoparticles encapsulated GA and reduced the release rate of GA, thus significantly delaying the escape of GA. On the other hand, due to the hydrogen bond interaction between NPs and GE, the coatings became denser, and the pathway in the spatial network structure of the GE coatings was blocked and hindered the escape of GA, thus further reducing the release rate of GA in the nanocomposite coatings. The results showed that CS nanoparticles encapsulated GA can significantly reduce the release rate of GA in GE coatings, effectively delay the escape of GA, and thus prolong the service life of GE coatings.

### 3.9. Mechanical and Self-Healing Properties

**Figure 5:**
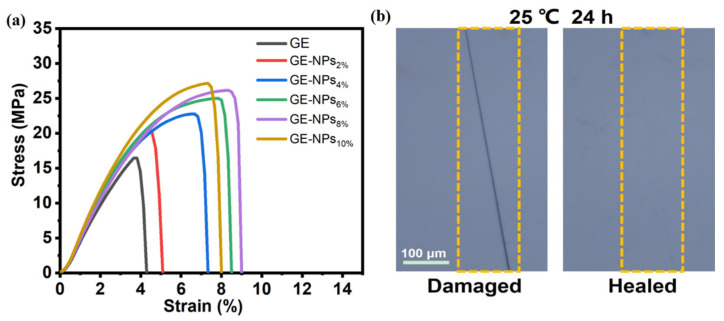
(**a**) Mechanical properties of the nanocomposite coatings; (**b**) The self-healing process of GE-NPs_10%_ coating was observed by the optical microscope.

The mechanical properties of the gelatin nanocomposite coatings were evaluated by tensile test at s strain rate of 100 mm min^−1^. Figure 5a shows the representative stress-strain curves of nanocomposite coatings with different NPs content. The introduction of NPs enhanced the stress and ductility of nanocomposite coatings, which may be attributed to the fact that the hydrogen bonds between NPs and gelatin can act as sacrificial bonds to dissipate energy for material toughening [45]. The self-healing ability of the coating helps to broaden its application field and ensure its reliability and stability [46]. The “scratch-repair” experiment was used to evaluate the self-healing performance of GE-NPs_10%_ coating. The scratch produced by the surgical blade on the coated surface can be repaired and restore the initial surface morphology within 24 h at 25 °C (Figure 5b).

## 4. Conclusions

In this study, NPs loaded with GA were added to GE coatings to prepare high-performance, degradable, self-healing bio-based nanocomposite coatings with antibacterial and antioxidant properties. When NPs was distributed in GE coating, the oxygen permeability coefficient of GE nanocomposite coatings decreased by 57.7%. At the same time, the light transmittance of the nanocomposite coatings at 300 nm decreased by 25%, which significantly enhanced the UV-blocking ability of the GE coating. What’s more, loading NPs can significantly improve the antioxidant activity of the nanocomposite coatings. When the addition of NPs was 10%, the antioxidant activity of the nanocomposite coatings increased by 119%. In addition, NPs endowed the GE coating with significant antibacterial properties, and the antibacterial rate against *E. coli* and *S. aureus* increased by 32% and 58%, respectively. Finally, encapsulating GA by CS nanoparticles can significantly delay the release of GA. After soaking in four simulated solutions (3% acetic acid, 50% ethanol, 95% ethanol, and water) for 24 h, the release rate of GA decreased by 94.1%, 95.7%, 89.31%, and 95.7%, respectively. Therefore, the GE coating loaded with NPs can be used as a potential antibacterial coating material.

## Data Availability

The data presented in this article are available on request from the corresponding author.

## Supplementary Materials

The following supporting information can be downloaded at: https://www.mdpi.com/article/10.3390/nano13071220/s1, Figure S1: Particle size distribution of chitosan nanoparticles loaded with GA; Figure S2: DTG spectrum of GE coating and GE-NPs10% coating; Table S1: Thermal degradation temperatures (Tpeak, °C) and weight loss (ΔW, %) of GE coating and GE-NPs10% coating.
