# Supplementary material for: High-Performance, Degradable, Self-Healing Bio-Based Nanocomposite Coatings with Antibacterial and Antioxidant Properties
Source: Nanomaterials (Basel). 2023 Mar 29;13(7):1220. doi: 10.3390/nano13071220 (PMC10096551; doi:10.3390/nano13071220)
Supplement: Supplementary file 1 [file nanomaterials-13-01220-s001.zip › nanomaterials-2257736-supplementary.pdf]

Supplementary Materials

High-Performance, Degradable, Self-Healing Bio-Based Nanocomposite Coatings with Antibacterial and Antioxidant Properties

Qiang Zhang [1,2,3], Qihang Bu [1,2,3], Jiangyue Xia [2,3], Rongxue Sun [2,3], Dajing Li [2,3], Haibo Luo [1,*], Ning Jiang [2,3,*] and Cheng Wang [2,3,*]

1 School of Food and Pharmaceutical Engineering, Nanjing Normal University, Nanjing 210023, China
2 Institute of Agricultural Products Processing, Jiangsu Academy of Agricultural Sciences, Nanjing 210014, China
3 Integrated Scientific Research Base for Preservation, Storage and Processing Technology of Aquatic Products of the Ministry of Agriculture and Rural Affairs, Nanjing 210014, China
* Correspondence: luohaibo_1216@126.com (H.L.); jaas_jiangning@163.com (N.J.); wangcheng@jaas.ac.cn (C.W.)

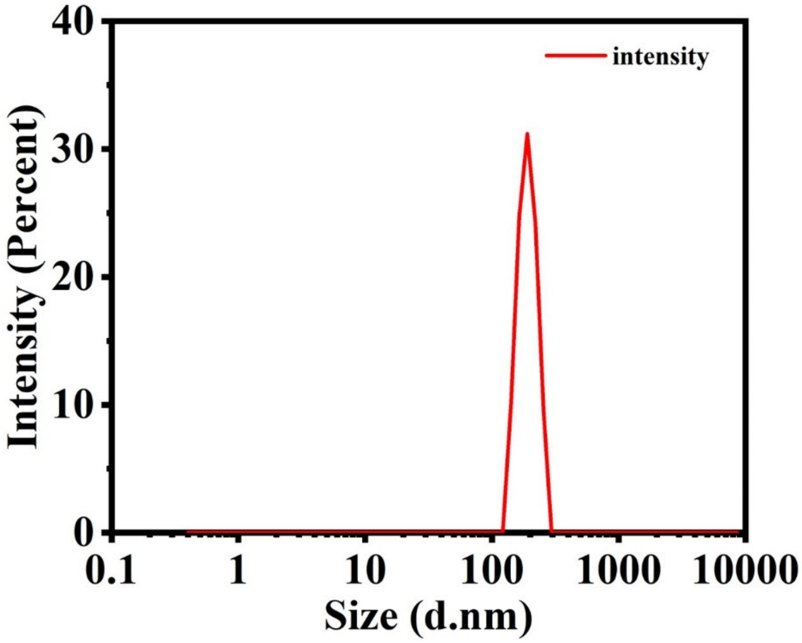

Figure S1. Particle size distribution of chitosan nanoparticles loaded with GA

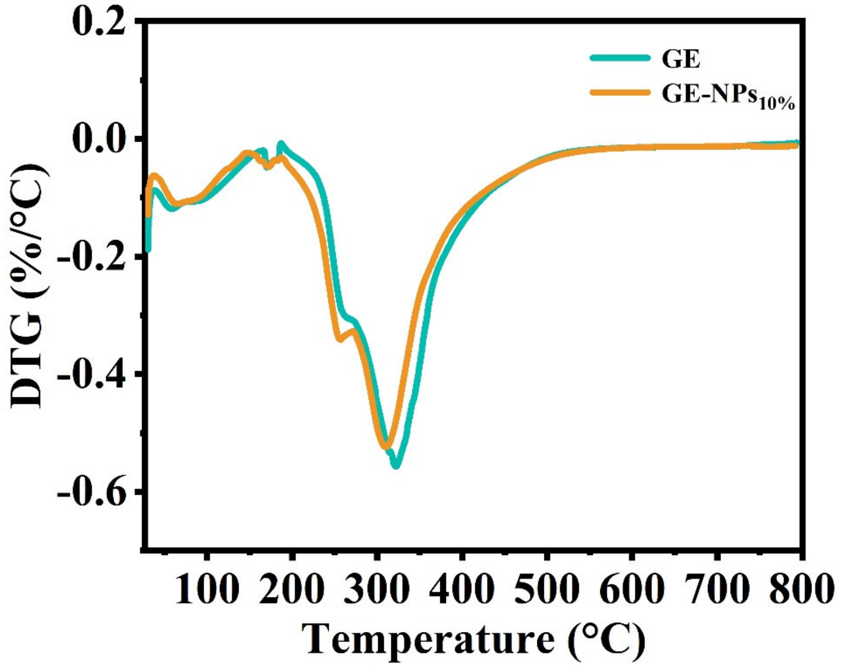

Figure S2. DTG spectrum of GE coating and GE-NPs10% coating

Table S1. Thermal degradation temperatures (Tpeak, °C) and weight loss (△W, %) of GE coating and GE-NPs10% coating

Sample	△1		△2		△3		Residue (%)
	Tpeak	△W1	Tpeak	△W2	Tpeak	△W3	
GE	55	13.5	262	12.2	322	51.3	18.8
GE-NPs10%	60	10.6	256	18.9	307	45	20.9

△1, △2 and △3 denote the first, second and third stage weight loss, respectively, of coating during heating scan.
